# Piezo2 Contributes to Traumatic Brain Injury by Activating the RhoA/ROCK1 Pathways

**DOI:** 10.1007/s12035-024-04058-y

**Published:** 2024-02-22

**Authors:** Yinggang Xiao, Yang Zhang, Wenjuan Yuan, Cunjin Wang, Yali Ge, Tianfeng Huang, Ju Gao

**Affiliations:** 1 Department of Anesthesiology, Northern Jiangsu People’s Hospital Affiliated to Yangzhou University/Clinical Medical College, Yangzhou University, Yangzhou, Jiangsu China; 2 Yangzhou Key Laboratory of Anaesthesiology, Yangzhou, Jiangsu China

**Keywords:** Traumatic brain injury, Piezo, Mechanobiological signal transduction, RhoA/ROCK1

## Abstract

Traumatic brain injury (TBI) can lead to short-term and long-term physical and cognitive impairments, which have significant impacts on patients, families, and society. Currently, treatment outcomes for this disease are often unsatisfactory, due at least in part to the fact that the molecular mechanisms underlying the development of TBI are largely unknown. Here, we observed significant upregulation of Piezo2, a key mechanosensitive ion channel protein, in the injured brain tissue of a mouse model of TBI induced by controlled cortical impact. Pharmacological inhibition and genetic knockdown of Piezo2 after TBI attenuated neuronal death, brain edema, brain tissue necrosis, and deficits in neural function and cognitive function. Mechanistically, the increase in Piezo2 expression contributed to TBI-induced neuronal death and subsequent production of TNF-α and IL-1β, likely through activation of the RhoA/ROCK1 pathways in the central nervous system. Our findings suggest that Piezo2 is a key player in and a potential therapeutic target for TBI.

**Supplementary Information:**

The online version contains supplementary material available at 10.1007/s12035-024-04058-y.

## Introduction

Traumatic brain injury (TBI) is a type of intracranial injury caused by direct or indirect forces acting on the head. According to the pathological changes that occur, TBI is divided into primary brain injury caused by structural disruption due to direct compression and displacement of brain tissue and consequent secondary brain injury, which involves a series of complex progressive changes such as breakdown of the blood‒brain barrier, the neuroinflammatory response, metabolic dysfunction, and neuronal death [1–3]. Currently, the main goal of treatment for TBI is to prevent primary injury and to block secondary damage [4]. Although a large amount of research has been conducted on the effects of various treatment strategies, such as inhibiting neuroinflammatory responses, correcting ion overload, alleviating oxidative stress, and restoring the blood‒brain barrier, the therapeutic effects of currently available treatments are still far from satisfactory [5]. The pathogenesis of TBI has become a hot topic in the fields of critical illness and neuroscience, but its pathogenesis is still far from fully understood, and there is a lack of effective treatment strategies.

Mechanical stimulation often accompanies TBI, and the conversion of mechanical information from mechanical stimuli or the surrounding environment into downstream biochemical reactions by neural cells and tissues is a complex cascade of events [6]. The main types of mechanical stimulation that can cause TBI are static hydrostatic pressure, shear force, and tensile or compressive force. Traumatic brain hematoma, edema, and other space-occupying lesion can develop in the areas surrounding brain tissue subjected to gradient static hydrostatic pressure [7, 8]. In addition, the space-occupying effect further increases intracranial pressure. All of these factors lead to an elevation of static hydrostatic pressure in brain tissue. The increase in hydrostatic pressure around neurons caused by brain edema due to TBI results in irreversible neuronal damage [6]. Moreover, during traumatic brain injury, blood from ruptured blood vessels enters the surrounding brain tissue, generating significant shear force and causing irreversible neuronal damage.

In recent years, a report in Science confirmed for the first time that Piezo family members are true mechanically sensitive ion channel proteins in mammals that mediate cytoskeleton reconstruction and stress-induced changes and play a key role in mechanical force transmission [9]. Of particular note is the recent discovery that neuronal axon growth not only is regulated by chemical signals but also depends on the regulation of mechanical force signals. Koser et al. [10] first reported in Nat Neurosci that Piezo can regulate the structure and growth direction of retinal ganglion cell (RGC) axons in *Xenopus laevis*. Knocking out the mechanically sensitive Piezo gene significantly changed the growth direction and structure of RGC axons. This strongly suggests that Piezo channel proteins with mechanical sensitivity in the brain may be involved in the occurrence and development of neurological disorders.

Piezo2 and Piezo1 are highly related in terms of structure [11–13]. Similar to Piezo1, the Piezo2 channel opens when the cell membrane is mechanically stimulated, leading to an influx of Ca^2+^ [14–16]. Ca^2+^ serves as the starting point for many biochemical reactions in cells, activating various downstream proteins and regulating gene expression, cellular cytoskeleton reorganization, and protein transportation [17–20]. Piezo2 is believed to interact with a range of intracellular Ca^2+^-response proteins [21]. This interaction can lead to specific outcomes, such as actin polymerization or the activation of transcription factors NFAT, Yap1, and β-catenin [18–20].

In this study, we established a controlled cortical impact (CCI) model in mice to simulate TBI and elucidated the role of mechanobiological signal transduction mediated by the neuronal mechanosensitive channel Piezo2 in TBI. The findings provide a potential target for the clinical treatment of TBI.

## Methods

### Experiment 1: Observation of Differences in Piezo2 Expression in Brain Tissues Between the TBI Group and Sham Group

Mice were divided into a Sham group and a TBI model group (TBI group). One day before modeling and 12 h and 1, 3, and 5 days after modeling, the neurological severity scores (NSSs) of the mice were evaluated. Each group underwent the Morris water maze (MWM) test on days 16–21. Western blotting (WB) was used to measure the expression of Piezo1 and Piezo2 in the brain tissues around the injury site and contralateral brain tissues at each time point mentioned above. Frozen brain tissue sections were prepared 3 days after TBI modeling, and single immunofluorescence staining was used to assess the expression of Piezo2, while double immunofluorescence staining was used for localization analysis of Piezo2 in microglia (Iba1), astrocytes (GFAP), and neurons (NeuN).

### Experiment 2: Evaluation of the Effects of Knocking Down or Inhibiting the Function of Piezo2 in TBI Mice and Changes in the Expression of the Downstream Molecules RhoA/ROCK1

The mice were divided into the sham surgery + scramble RNA negative control (Sham + NC), model + shRNA negative control (TBI + NC), model + Piezo2-shRNA (TBI + shRNA), Sham surgery + Piezo2-shRNA (Sham + shRNA), Sham + vehicle, TBI + vehicle, TBI + D-GsMTx4, and Sham + D-GsMTx4 groups. At 3 days after TBI modeling, the NSSs of the mice were determined, followed by HE staining and Nissl staining of frozen brain tissue sections. All groups underwent the MWM test on days 16–21. WB was used to measure the protein expression of Piezo2, Iba1, GFAP, IL-1β, TNF-α, ROCK1, total RhoA, and RhoA-GTP in the brain tissue surrounding the injury in each group. Three days after modeling, double immunofluorescence staining for Piezo2 and ROCK1 or RhoA was performed.

#### Experimental Animals

A total of 154 healthy clean-grade C57BL/6 J mice aged 8–12 weeks and weighing 21–24 g were provided by the Animal Experimental Center of Yangzhou University and randomly assigned to groups using a random number table. The mice were housed in clean animal rooms with adequate food and water on a 12-h light/dark cycle.

#### Construction of a Traumatic Brain Injury Mouse Model

Mice were anesthetized with 5% isoflurane, and anesthesia was maintained with 2% isoflurane. The mouse’s head was fixed in a stereotactic frame, and a constant temperature blanket was used to maintain the body temperature at 37.0 ± 0.5 °C. A midline incision was made to expose the coronal suture, sagittal suture, and bilateral coronal ridges. A 4-mm-diameter hole was drilled in the right coronal ridge, with the center of the hole located being between the coronal suture and the coronal ridge, while keeping the dura mater intact. CCI injury was induced as previously described [22]. A PinPoint Precision Cortical Impactor PCI3000 (Hateras) with a 3-mm-diameter cylindrical impactor was used to strike the cortical surface vertically. The impact parameters used in this experiment were as follows: speed, 1.5 m/s; impact depth, 1.5 mm; and duration, 100 ms. After impact, the cortex was covered with sterile cotton, and the skin was sutured with 6–0 silk. Body temperature was maintained at 37.0 ± 0.5 °C throughout the experiment until full consciousness was restored. In the Sham group, the scalp was incised, and the skull was exposed; however, the brain was not impacted.

#### shRNA and D-GsMTx4 Injection

pAAV-U6-shRNA(Piezo2)-CMV-MCS-WPRE and pAAV-U6-shRNA(NC)-CMV-MCS-WPRE were designed and synthesized by Obio Technology Corporation (Shanghai, China). The Piezo2-siRNA sequence was CCTCTTCTTGTTTCAAGGGTT, and the NC-siRNA sequence was CCTAAGGTTAAGTCGCCCTCG. Injection was performed after the plasmid was packaged into an adeno-associated virus vector. D-GsMTx4 (Tocris) was dissolved in saline to a concentration of 5 μg/μl (for WB and slice staining), 10 μg/μl, or 15 μg/μl. Ten minutes after modeling, the left lateral cerebral ventricle was located using a brain stereotaxic apparatus and the following coordinates: 0.5 mm posterior to bregma, 1 mm left of bregma, and 2.5 mm below the skull surface. A hole was slowly drilled using a microperforated dental drill, and a fixed microinjector was used to inject 300 nl of the appropriate shRNA solution, 1 μl of D-GsMTx4, or an equivalent amount of scrambled shRNA or saline into each mouse. The injection time was 5 min, and the needle was left in place for 10 min after injection to ensure full absorption of the solution. The needle was slowly withdrawn, the drilling site was coated with bone wax, and the scalp was sutured.

#### NSSs

NSSs were used to evaluate the mice’s motor (muscle strength and abnormal movements), sensory (vision, touch, and balance), and reflex function, as described previously [23]. TBI modeling was considered successful for mice with an NSS more than 2 and 5 on the first day after modeling, with reference to a previous study [24]. A point was awarded when a mouse failed to complete a task or exhibited loss of a reflex. A higher score indicates more severe nerve damage.

#### MWM Test

Referring to the study of Ge et al. [25], the maze consisted of a circular tank with a diameter of 120 cm and a height of 50 cm that was filled with water dyed white with nontoxic titanium dioxide dye; the water temperature was kept at 24–26 °C, and the pool was surrounded by blue blackout curtains. The pool was divided into four quadrants, and in the fourth quadrant, there was a movable circular platform, 15 cm in diameter, submerged approximately 1 cm below the surface. The positioning navigation training phase was performed at 8:00 am every day from the 16th to 20th day after TBI; the mice were placed in the water from different quadrants for training. They were given 60 s to find the platform and were allowed to stayed on the platform for 5 s. If the platform was not found within 60 s, the mouse was guided to the platform and allowed to stay there and learn its location for 30 s. ANY-maze (Stoelting, USA) was used to obtain video recordings and data and calculate the latency of animals to reach the platform (escape latency). In the spatial exploration phase, which was performed on the 21st day after TBI modeling, the platform was removed, and the mice were placed in the third quadrant, which was farthest from the original platform location. The time the mice spent in the target quadrant, the number of times they crossed the platform, and their swimming speed over 60 s were recorded for each group.

#### WB

WB was used to measure target protein expression. Brain tissue surrounding the injury or matching brain tissue from mice without TBI was collected and homogenized in RIPA lysis buffer with PMSF. The sample was centrifuged at 12,000 rpm for 10 min at 4 °C, the supernatant was collected, and the protein concentration was quantified using the BCA method. Equal amounts of protein were separated by 8% SDS‒PAGE and then transferred to nitrocellulose membranes by wet transfer. After blocking with 5% skim milk for 2 h, the membranes were probed with rabbit anti-Piezo2 (Thermo Scientific, 1:500, Cat. Number: PA5-72,976), rabbit anti-Piezo1 (Thermo Scientific, 1:500, Cat. Number: MA5-32,876), rabbit anti-Iba-1 (Thermo Scientific, 1:2000, Cat. Number: PA5-27,436), rabbit anti-GFAP (Thermo Scientific, 1:1000, Cat. Number: PA5-16,291), rabbit anti-TNF-α (Cell Signaling Technology, 1:1000, Cat. Number: # 3707S), rabbit anti-IL-1β (Thermo Scientific, 1:1000, Cat. Number: P420B), rabbit anti-ROCK1 (Thermo Scientific, 1:3000, Cat. Number: PA5-22,262), rabbit anti-phospho-RhoA (Thermo Scientific, 1:2000, Cat. Number: PA5-105,763), rabbit anti-RhoA (1:1500, Cat. Number: PA5-87,403), and rabbit anti-GAPDH (Santa Cruz, 1:3000, Cat. Number: sc-365062) antibodies overnight at 4 °C. After washing with PBST, the membranes were probed with goat anti-rabbit IgG secondary antibody (Jackson ImmunoResearch, 1:3000, Cat. Number: 111–005-003) at room temperature for 2 h, and the protein bands were visualized using an enzyme-based detection system followed by scanning. The expression level of the target protein was calculated as the ratio of the gray value of the target protein band to that of the GAPDH band using ImageJ.

#### HE Staining and Nissl Staining

Mice were anesthetized with 5% isoflurane, and the thorax was exposed. The right atrium was cut, and PBS (40 ml) and 4% PFA (40 ml) were sequentially perfused through the left ventricle. The brain tissue was fixed in 4% PFA at 4 °C overnight and dehydrated with 15% and 30% sucrose for 24 h each, and frozen Sects. (30 μm) were obtained and subjected to HE kit (Servicebio) and Nissl staining by 0.5% toluidine blue (Servicebio) according to the instructions [26, 27]. The samples were observed and photographed under a microscope (Leica). In ImageJ, Nissl bodies were counted by randomly selecting sections at the same site from each mouse.

#### Immunofluorescence Staining

Immunofluorescence staining was performed according to the methods previously reported by our research group [28]. Frozen sections were blocked with PBS containing 5% goat serum and 0.3% Triton X-100 at 37 °C for 1 h and then incubated with a mixture of rabbit polyclonal anti-Piezo2 antibody (Thermo Scientific, 1:200, Cat. Number: PA5-72,976), goat polyclonal anti-NeuN antibody (Thermo Scientific, 1:500, Cat. Number: PA5-143,586), goat polyclonal anti-GFAP antibody (Thermo Scientific, 1:500, Cat. Number: PA5-143,587), goat polyclonal anti-Iba1 antibody (FUJIFILM Wako Chemicals, 1:1000, Cat. Number: 011–27991), mouse monoclonal anti-ROCK1 antibody (Thermo Scientific, 1:500, Cat. Number: MA5-27,778), and mouse monoclonal anti-RhoA antibody (Thermo Scientific, 1:500, Cat. Number: MA1-134) at 4 °C overnight. After washing, the sections were incubated with corresponding secondary antibodies, including Cy3-labeled goat anti-rabbit IgG (Jackson ImmunoResearch, 1:500, Cat. Number: 111–165-003), Cy2-labeled donkey anti-goat IgG (Jackson ImmunoResearch, 1:500, Cat. Number: 705–225-147), Cy2-labeled goat anti-rabbit lgG (Jackson ImmunoResearch, 1:500, Cat. Number: 111–225-144), and Cy3-labeled donkey anti-mouse IgG (Jackson ImmunoResearch, 1:500, Cat. Number: 715–165-150), at room temperature for 1 h. Finally, the sections were counterstained with DAPI for 1 min to label the cell nuclei. All sections were observed and photographed using a Leica DMI4000 fluorescence microscope. Cells labeled with a single probe were counted using ImageJ.

#### Statistical Analysis

SPSS 23.0 was used for statistical analysis. All data are presented as the mean ± standard deviation. One-way analysis of variance (ANOVA) or two-way repeated measures ANOVA followed by Bonferroni’s post hoc test was used for comparisons among multiple groups, while two independent samples were compared by two-tailed independent sample *t* test. *P* < 0.05 was considered to indicate statistical significance.

## Result

### Piezo2 Protein Exhibited Increased Expression After TBI and Localized to Neurons

**Fig. 1:**
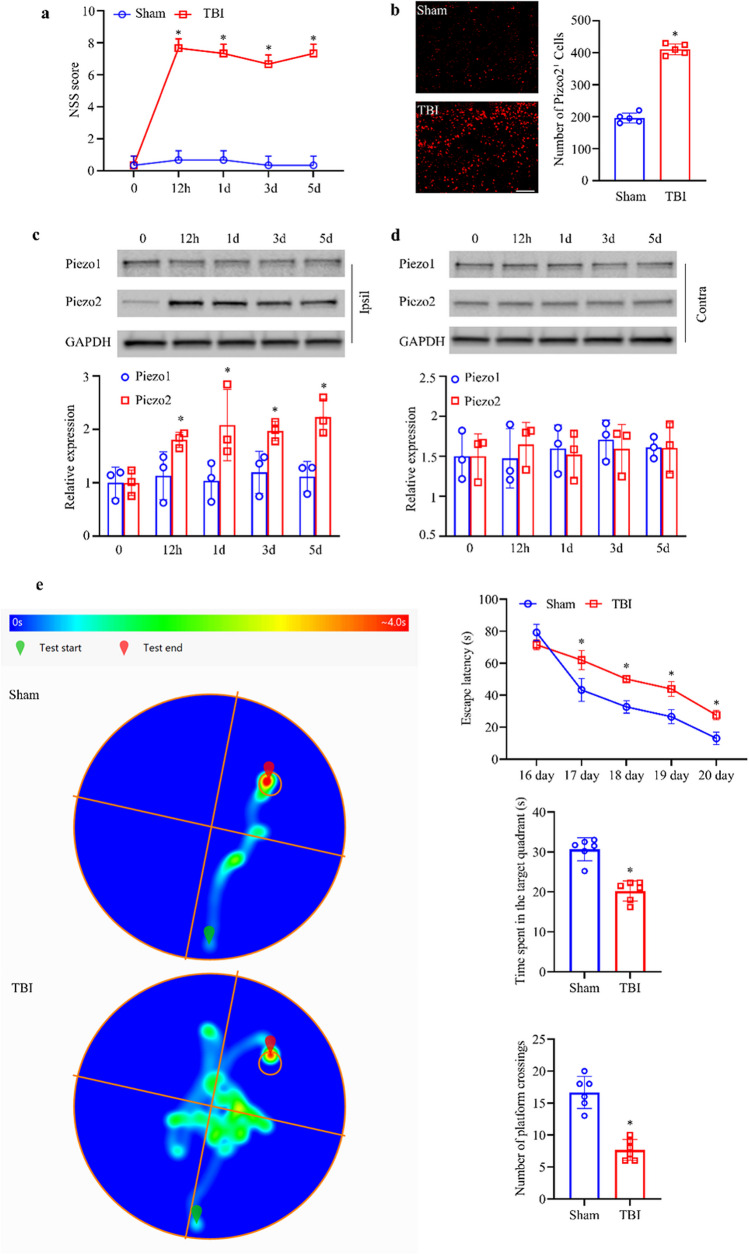
Effect of CCI. **a** Effect of CCI on NSSs. *n* = 3 mice/group. **b** Piezo2 immunofluorescence staining in the ipsilateral brain on day 3 after CCI or Sham surgery. Scale bar, 100 μm. Statistical summary of the densitometric data (right). *n* = 5 biological replicates/group. **c**, **d** Levels of Piezo1 and Piezo2 protein in ipsilateral (Ipsi) and contralateral (Contra) brain tissue on different days in the two groups. *n* = 3 biological replicates/group/time point. **e** MWM. The left is a heat map of mouse movement trajectories, and the right is escape latency, time spent in the target quadrant, and the number of platform crossings, listed from top to bottom. *n* = 6 mice/group. **a**–**e** *P < 0.05 versus the Sham group; two-tailed unpaired Student’s *t* test

**Fig. 2:**
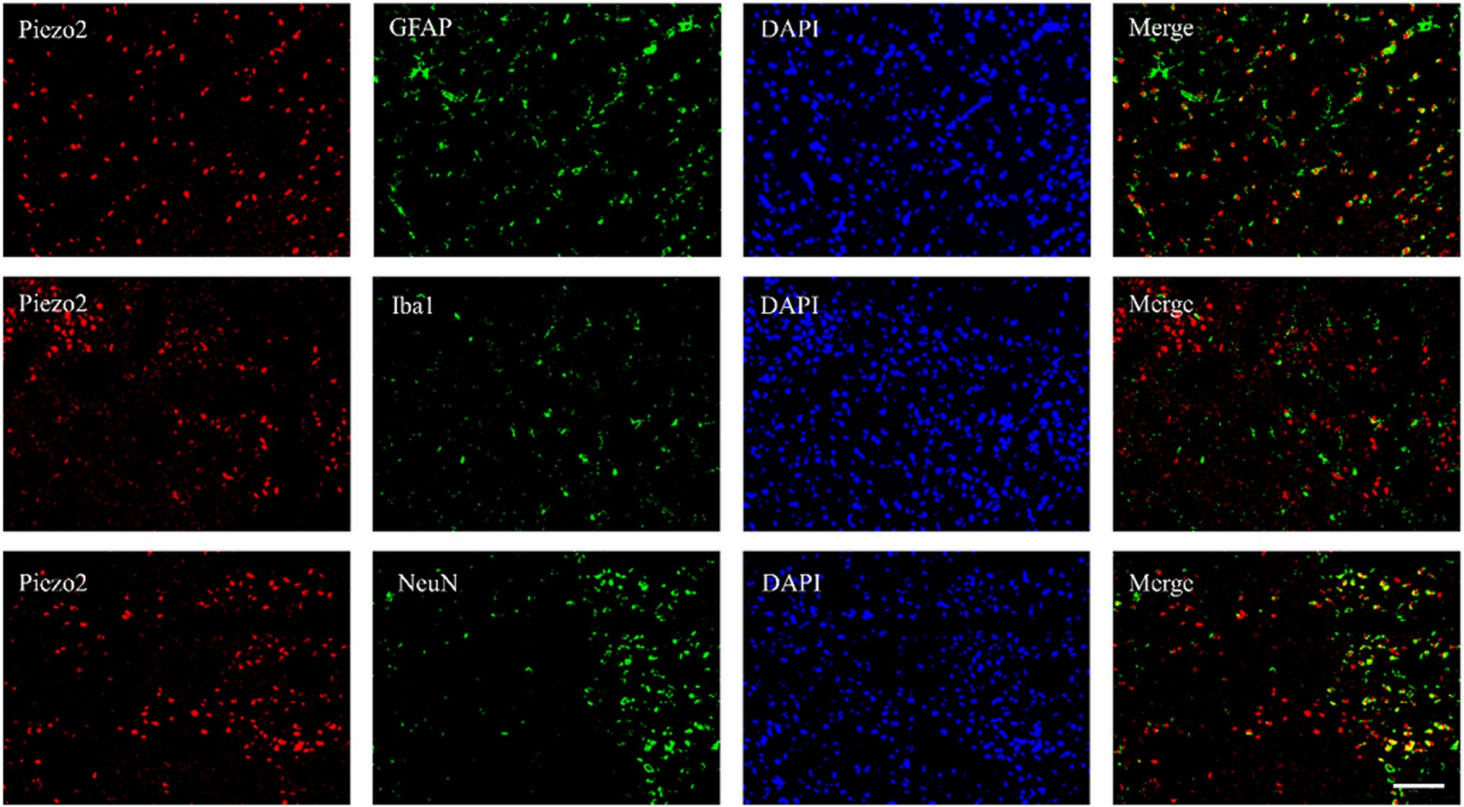
Double immunofluorescence staining for Piezo2, Iba1, GFAP, and NeuN in the ipsilateral brain on day 3 post-CCI. Representative samples from three biological replicates are shown. Scale bar, 100 μm

After TBI, mice exhibit significant and sustained neurological damage. Neurological impairment appeared at 12 h post-modeling and lasted for at least 5 days (Fig. 1a). The escape latency of mice in the TBI group was higher than that of mice in the control group on the second to fifth day of the first phase of the water maze test; on the sixth day, the TBI mice stayed in the fourth quadrant, where the platform had been previously located, significantly longer than the mice in the control group, and the number of platform crossings was significantly higher in the TBI group than in the control group (Fig. 1e); there was no significant difference in swimming speed between the two groups (Supplementary Fig. 1a). We also examined the changes in the expression of Piezo family members after TBI. Immunofluorescence staining showed a significant increase in Piezo2 expression in the brain tissue surrounding the damaged area (Fig. 1b). Piezo2 expression was significantly upregulated on the injured side of the brain in TBI mice from 12 h, and this change lasted for at least 5 days; however, there was no significant change in Piezo1 expression on either side of the brain in the TBI group (Fig. 1 c and d). To further determine the cellular localization of Piezo2, double immunofluorescence staining was performed on slices of brain tissue from around the damaged area. Piezo2 was mainly expressed in neurons, with a small number of astrocytes and microglia also expressing Piezo2 (Fig. 2). TBI caused upregulation of Piezo2 expression but not Piezo1 expression in mice, which persisted higher than in normal mice. Increased Piezo2 was mainly expressed in neurons.

#### Piezo2-shRNA Microinjection Attenuated TBI

**Fig. 3:**
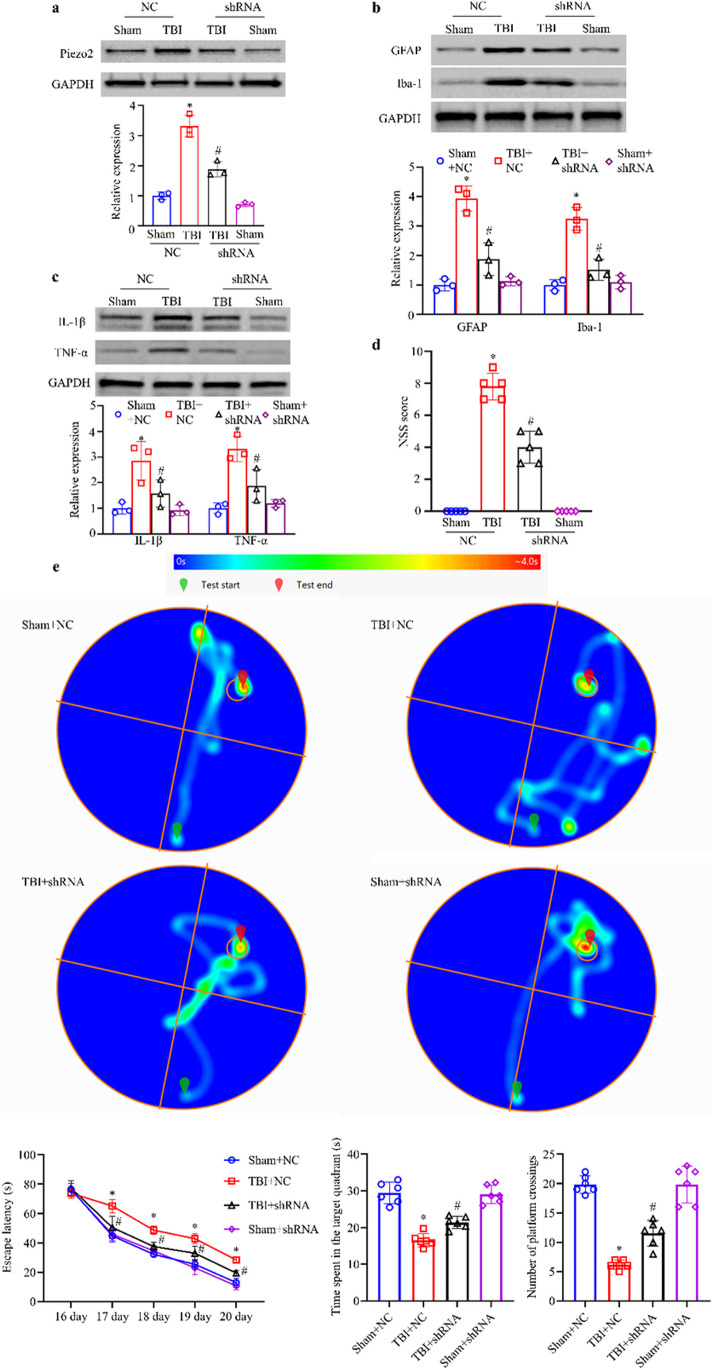
Effect of microinjection of Piezo2-shRNA. **a** Expression of Piezo2. **b** Expression of Iba-1 and GFAP. **c** Expression of IL-1β and TNF-α. All the images are of brain tissue from the ipsilateral side. Top: representative Western blots. Bottom: statistical summary of the densitometric data. n = 3 biological replicates/group. **d** NSSs. *n* = 5 mice/group. **e** MWM. The top is a heat map of mouse movement trajectories, and the bottom is escape latency, time spent in the target quadrant, and the number of platform crossings, listed from left to right. *n* = 6 mice/group. **a**–**e** One-way ANOVA followed by Bonferroni’s post hoc test. *P < 0.05 versus the Sham + NC group. ^#^*P* < 0.05 versus the TBI + NC group

We further explored whether shRNA-mediated inhibition of TBI-induced Piezo2 upregulation could alleviate brain damage. At 12 h after modeling, the NSS of the TBI + shRNA group was significantly lower than that of the TBI + NC group, indicating reduced neurological damage (Fig. 3d). Compared with the TBI + NC group, the TBI + shRNA group exhibited a shorter escape latency, longer time spent in the target quadrant, and increased number of platform crossings (Fig. 3e). Meanwhile, there was no significant difference in swimming speed among all the groups (Supplementary Fig. 1b). The WB results showed that Piezo2-shRNA significantly inhibited the upregulation of Piezo2, GFAP, and Iba-1 by TBI but did not completely reverse the changes (Fig. 3a and b). After Piezo2 expression was inhibited, the upregulation of proinflammatory factors by TBI was significantly suppressed. The expression levels of IL-1β and TNF-α in the TBI + shRNA group were significantly lower than those in the TBI + NC group (Fig. 3c). Under light microscopy, it was observed that TBI caused massive necrosis of cortical neurons in the damaged area, the cells in the surrounding brain tissue were loosely arranged, and intercellular edema was evident. Nuclei were condensed, and the cytoplasm was loosely arranged, exhibited lighter staining, and showed vacuolization. Nissl staining showed that the number of Nissl bodies in TBI + NC group was decreased, and Nissl staining was lighter in this group, indicating severe neuronal degeneration. Piezo2-shRNA, however, reduced the degree of damage to the lesion site and its surroundings (Supplementary Fig. 2a and b). These results suggest that Piezo2-shRNA downregulated Piezo2 expression in TBI mice and that Piezo2 knockdown reduces neuronal degeneration, inflammation, and death, as well as the degree of brain edema, and protects normal neurological function in mice to a certain extent.

#### Pretreatment with D-GsMTx4 Attenuated TBI

**Fig. 4:**
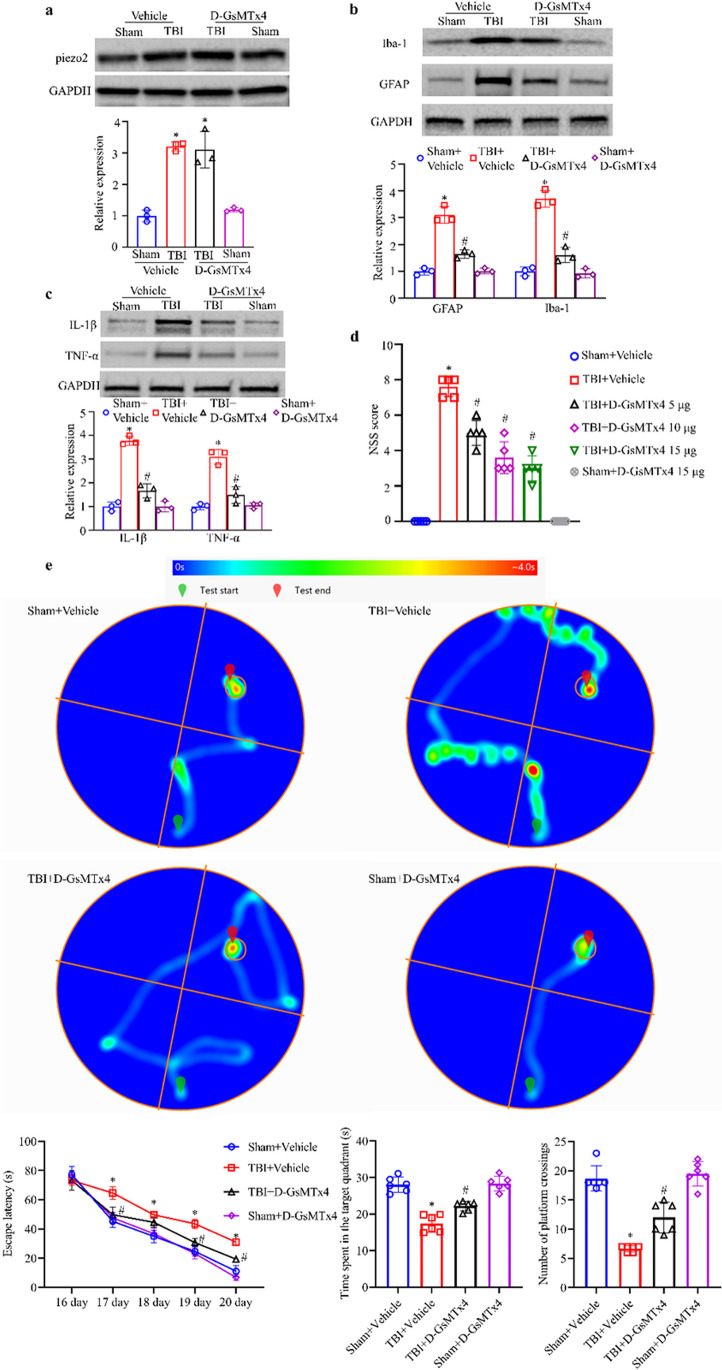
Effect of microinjection of D-GsMTx4. **a** Expression of Piezo2. **b** Expression of Iba-1 and GFAP. **c** Expression of IL-1β and TNF-α. All the images are of brain tissue from the ipsilateral side. Top: representative Western blots. Bottom: statistical summary of the densitometric data. *n* = 3 biological replicates/group. One-way ANOVA followed by Bonferroni’s post hoc test. **d** NSSs. *n* = 5 mice/group. One-way ANOVA followed by Bonferroni’s post hoc test. **e** MWM. The top is a heat map of mouse movement trajectories, and the bottom is escape latency, time spent in the target quadrant, and the number of platform crossings, listed from left to right. *n* = 6 mice/group. **a**–**e** One-way ANOVA followed by Bonferroni’s post hoc test. *P < 0.05 versus the Sham + vehicle group. ^#^*P* < 0.05 versus the TBI + vehicle group

To verify whether Piezo2 aggravates TBI by increasing the intracellular calcium ion concentration and activating downstream pathways, we injected 5 µg D-GsMTx4 before TBI modeling to inhibit Piezo2 function. D-GsMTx4 did not change the expression levels of Piezo2 in the TBI or Sham group (Fig. 4a) but significantly suppressed the upregulation of GFAP and Iba-1 induced by TBI (Fig. 4b) and the increase in the expression of the proinflammatory factors IL-1β and TNF-α (Fig. 4c). NSSs showed that D-GsMTx4 improved mouse neurological function after TBI in a concentration-dependent manner, and the high concentration of D-GsMTx4 (15 μg) did not show neurotoxicity in mice (Fig. 4d). Compared with the TBI + vehicle group, the TBI + D-GsMTx4 group exhibited a shorter escape latency, longer time spent in the target quadrant, and increased number of platform crossings (Fig. 4e). Meanwhile, there was no significant difference in swimming speed among all the groups (Supplementary Fig. 1c). HE staining and Nissl staining results showed that D-GsMTx4 partially reversed brain tissue damage, neuroinflammation, and neuronal death caused by TBI (Supplementary Fig. 3a and b). These results further prove that Piezo2 activation is a key process in the development of TBI.

#### The Increase in Piezo2 Expression Activated RhoA/ROCK1 in the Brains of TBI Mice

**Fig. 5:**
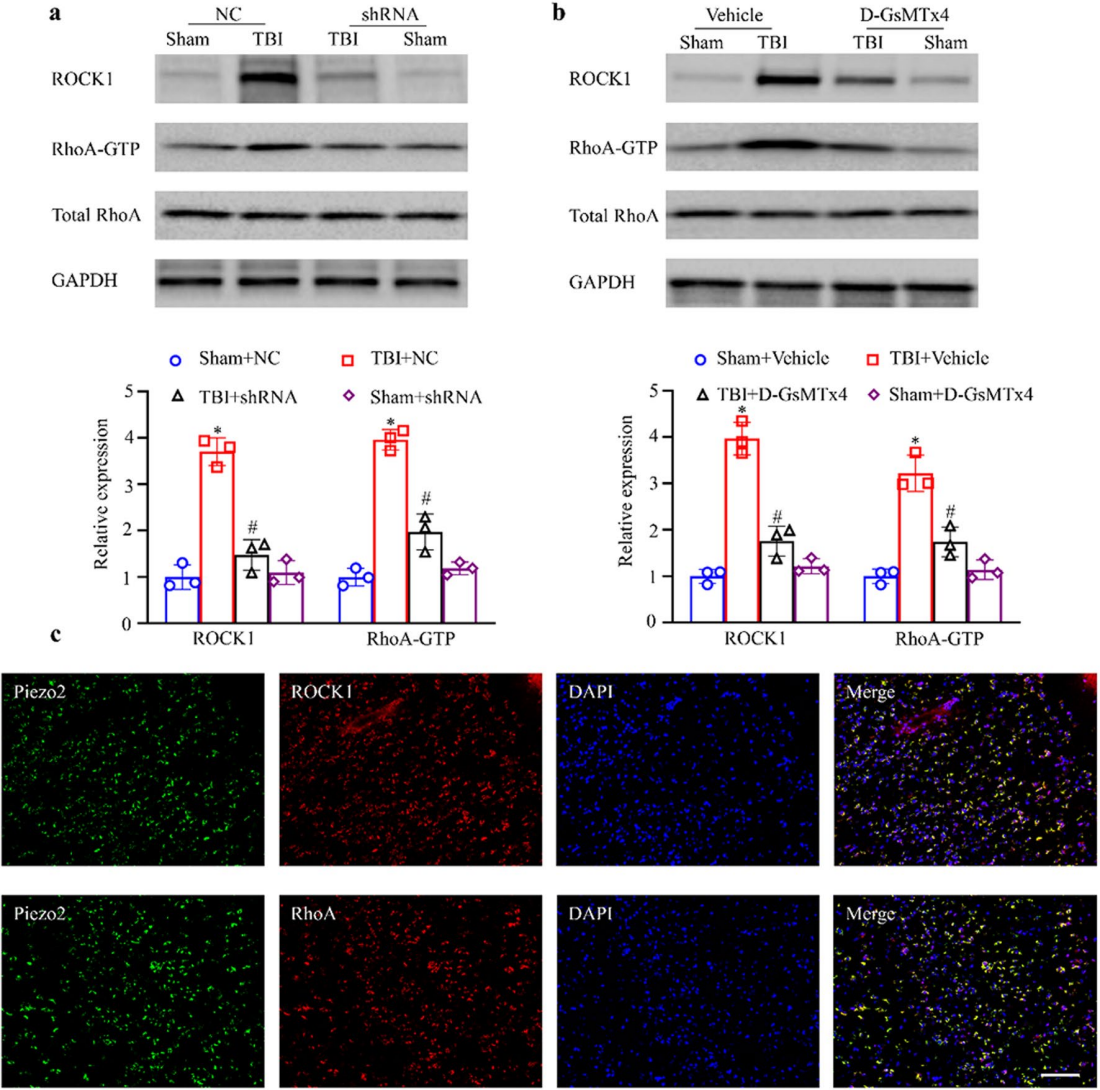
Effect of Piezo2 on the RhoA/ROCK1 pathway. **a** Effect of Piezo2-shRNA on the levels of total RhoA, RhoA-GTP, and ROCK1. *n* = 3 biological replicates/group. One-way ANOVA followed by Bonferroni’s post hoc test. *P < 0.05 versus the Sham + NC group. ^#^*P* < 0.05 versus the TBI + NC group. **b** Effect of D-GsMTx4 on the expression of Iba1 and GFAP. All the images are of brain tissue from the ipsilateral side. Top: representative Western blots. Bottom: statistical summary of the densitometric data. *n* = 3 biological replicates/group. One-way ANOVA followed by Bonferroni’s post hoc test. *P < 0.05 versus the Sham + vehicle group. ^#^*P* < 0.05 versus the TBI + vehicle group. **c** Double immunofluorescence staining for Piezo2, RhoA, and ROCK1 in the ipsilateral brain on day 3 post-CCI. Representative samples from three biological replicates are shown. Scale bar, 100 μm

To explore downstream targets of Piezo2 in the development of TBI, we studied the expression levels of proteins in the RhoA/ROCK1 signaling pathway. WB results showed that there was no significant difference in the expression level of total RhoA among the groups, but the levels of activated RhoA-GTP and its downstream target ROCK1 were significantly higher in the TBI + NC group than in the Sham + NC group; moreover, RhoA-GTP/ROCK1 levels in the TBI + shRNA group were significantly lower than that in the TBI + NC group (Fig. 5a). D-GsMTx4 also inhibited the significant increase in RhoA-GTP/ROCK1 levels in brain tissue after TBI (Fig. 5b). Double immunofluorescence staining showed that Piezo2 and RhoA/ROCK1 were coexpressed on the cell membrane (Fig. 5c). These results indicate that the RhoA/ROCK1 signaling pathway is activated after TBI and may be regulated by the increase in intracellular calcium ion concentrations caused by Piezo2.

## Discussion

The Piezo family currently contains two members, Piezo1 and Piezo2. Piezo1 is mainly expressed in tissues such as the lungs, bladder, and skin, while Piezo2 is mainly expressed in nervous tissues [9]. After sensing mechanical stimulation, Piezo proteins can participate in regulating a variety of physiological processes, including cell migration and differentiation, by mediating Ca^2+^ influx [29–31]. Research has found that Piezo2 channels in neuronal cells can convert mechanical stimuli into Ca^2+^-mediated action potentials. Specific knockout of the Piezo2 gene in neuronal cells results in the disruption of the response to mechanical stress [32]. A cell study found that knocking out Piezo2 can reduce vascular endothelial growth factor (VEGF)- or interleukin-1β (IL-1β)-mediated vascular leakage and can cause changes in intracellular Ca^2+^ and ATP concentrations, inhibition of Wnt11/β-catenin signaling, and disruption of vascular endothelial cell tight junction formation and vascular genesis [33]. Notably, a recent study using a TBI model found that blast shock waves can cause neuronal death, inflammatory reactions in brain tissue, and damage to the blood‒brain barrier. It was particularly found that the expression of the Piezo2 protein in brain tissue significantly increased and that the expression of the Piezo2 protein was significantly correlated with the severity of brain injury caused by blast shock waves [34]. However, the authors did not investigate the key role of Piezo2 in the pathophysiological changes in this model or the related molecular mechanism.

Our research found that Piezo2, but not Piezo1, plays a key role in TBI. Piezo2 expression increases continuously after TBI, and double immunofluorescence staining showed that this increase mainly occurs in neurons rather than astrocytes and microglia. However, it is still unknown how Piezo2 is activated after TBI. Epigenetic modifications, changes in transcription factor expression, and increased mRNA stability are potential mechanisms that require further study. Our study also found that the release of inflammatory factors, neuronal death, and neuronal function impairment after TBI can be alleviated not only by Piezo2-shRNA but also by the Piezo2-specific inhibitor D-D-GsMTx4, although the effects were not completely reversed. In addition, the effect of the inhibitor was dose dependent. D-D-GsMTx4 can effectively inhibit the opening of the Piezo2 ion channel in the cell membrane and reduce cation influx [35].

The Ras homolog gene family protein A (RhoA)/Rho-associated coil-coil containing protein kinase (ROCK) pathway is involved in various physiological activities of cells, including cytoskeleton reconstruction, contraction, migration, phagocytic adhesion, stress fiber formation, the inflammatory response, and angiogenesis, and recent studies have found it to be closely related to the polarization of microglia [36, 37]. Targeting the inhibition of the RhoA/ROCK signaling pathway has gradually become the most promising direction for the treatment of TBI, and multiple molecules, such as Nogo, CSPG, and glutamate, may be upstream molecules of this pathway [38]. A key study reported that spatial cognition can be improved after TBI by inhibiting ROCK1 upregulation [39]. These findings confirm the role of RhoA/ROCK1 in mediating TBI; however, the upstream mechanism responsible for regulating mechanical information remains unclear and requires further study.

The RhoA protein, as a small G protein, is also regulated by guanine nucleotide exchange factors (GEFs). In the resting state, RhoA binds to GDP and is biologically inactive, but after being catalyzed by GEFs, RhoA then binds to GTP and is converted to an activated state [40]. Our study found that although Piezo2-shRNA and D-D-GsMTx4 did not change the expression level of RhoA, they inhibited the activation of RhoA and downregulated the expression of ROCK1. In addition, the expression of Piezo2 and RhoA/ROCK1 was increased in the same cells in TBI mice, which further proves that the RhoA/ROCK1 pathway is a key signaling pathway mediated by Piezo2 in TBI. However, whether the increase in the levels of the proinflammatory cytokines IL-1β and TNF-α is related to the activation of the RhoA/ROCK1 pathway still needs further study.

In conclusion, our study shows, for the first time, that Piezo2 mediates the inflammatory response and neuronal degeneration and death in brain tissue and neuronal function after TBI through the use of both drug inhibitors and gene knockdown approaches. Piezo2 may be a potential target for the clinical treatment of TBI. However, Piezo2 may also be expressed in other systems and organs and participate in critical pathophysiological processes, which is one of the challenges for the broad clinical application of its inhibitors. In addition, it is worth noting that further clarification of the upstream regulatory mechanism of Piezo2 can help to elucidate the role of mechanobiological signal transduction in the pathophysiological process of TBI.

## Supplementary Information

Below is the link to the electronic supplementary material.

Supplementary file1 (DOCX 4727 KB)

## Data Availability

Not applicable.
