# Supplementary material for: Piezo2 Contributes to Traumatic Brain Injury by Activating the RhoA/ROCK1 Pathways
Source: Mol Neurobiol. 2024 Feb 22;61(10):7419–30. doi: 10.1007/s12035-024-04058-y (PMC11415480; doi:10.1007/s12035-024-04058-y)
Supplement: Supplementary file 1 — Supplementary file1 (DOCX 4727 KB) [file 12035_2024_4058_MOESM1_ESM.docx]

**Supplementary Materials**


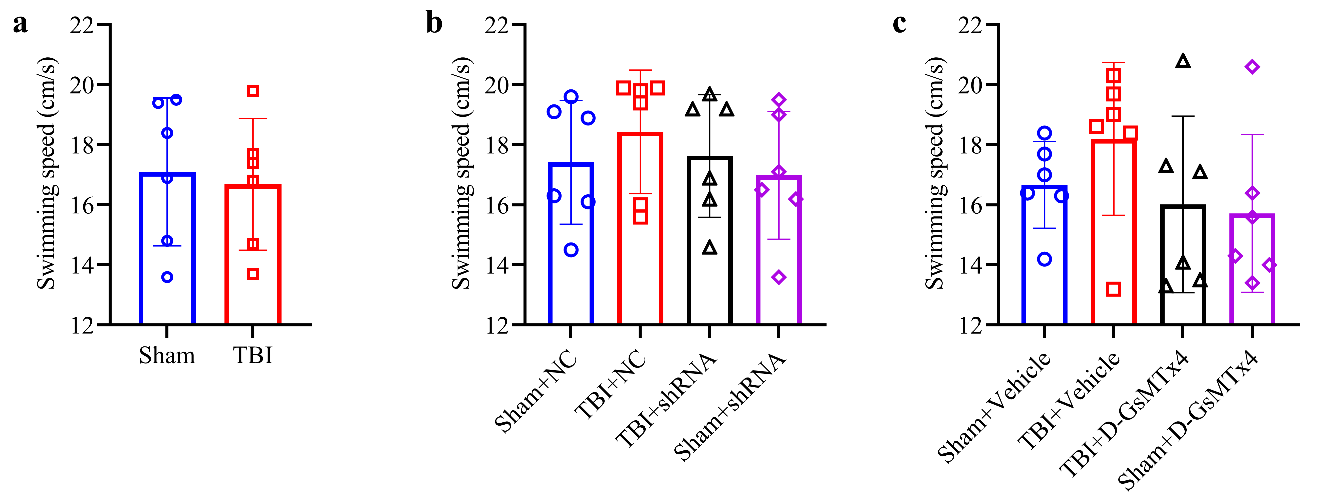


**Supplementary Fig. 1** Mouse swimming speed in the MWM. (a) Effect of CCI. *P < 0.05 versus the Sham group; two-tailed unpaired Student’s t test. (b) Effect of microinjection of Piezo2-shRNA. One-way ANOVA followed by Bonferroni’s post hoc test. *P < 0.05 versus the Sham + NC group. #P < 0.05 versus the TBI + NC group. (c)Effect of microinjection of D-GsMTx4. One-way ANOVA followed by Bonferroni's post hoc test. *P < 0.05 versus the Sham + vehicle group. #P < 0.05 versus the TBI + vehicle group. (a-c) n = 6 mice/group


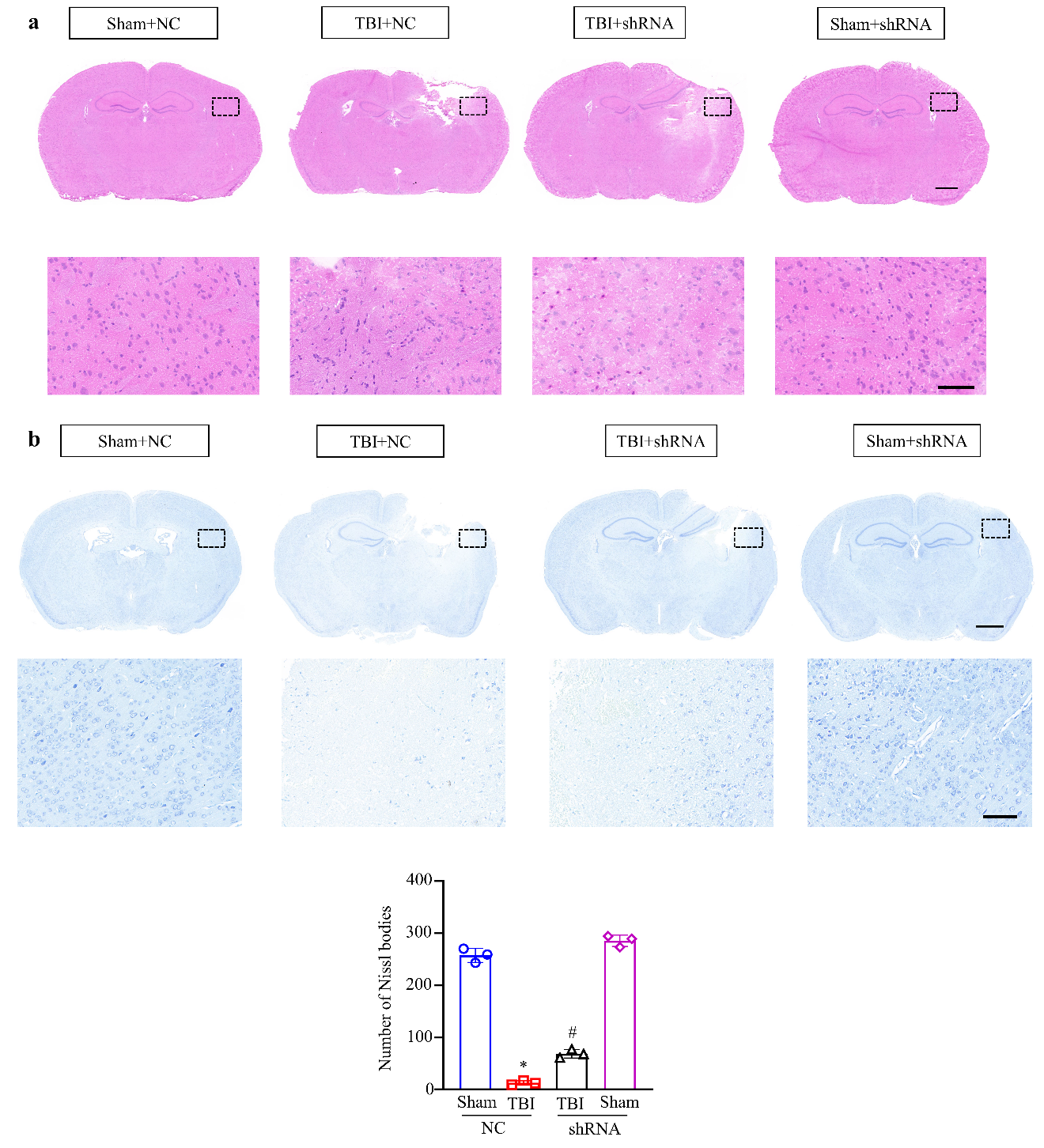


**Supplementary Fig. 2** Effect of administration of Piezo2-shRNA or scrambled shRNA on brain tissue on day 3. (a) Representative HE-stained brain sections from the different treatment groups. Top: images of entire brain sections. Scale bar: 1000 µm. Bottom: magnification of the photographs in the top panel. Scale bar: 100 µm. (b) Representative Nissl-stained brain sections from the different treatment groups. Top: images of entire brain sections. Scale bar: 1000 µm. Bottom: magnification of the photographs in the top panel. Scale bar: 100 µm. n=3. One-way ANOVA followed by Bonferroni’s post hoc test. *P < 0.05 versus the Sham + NC group. #P < 0.05 versus the TBI + NC group.


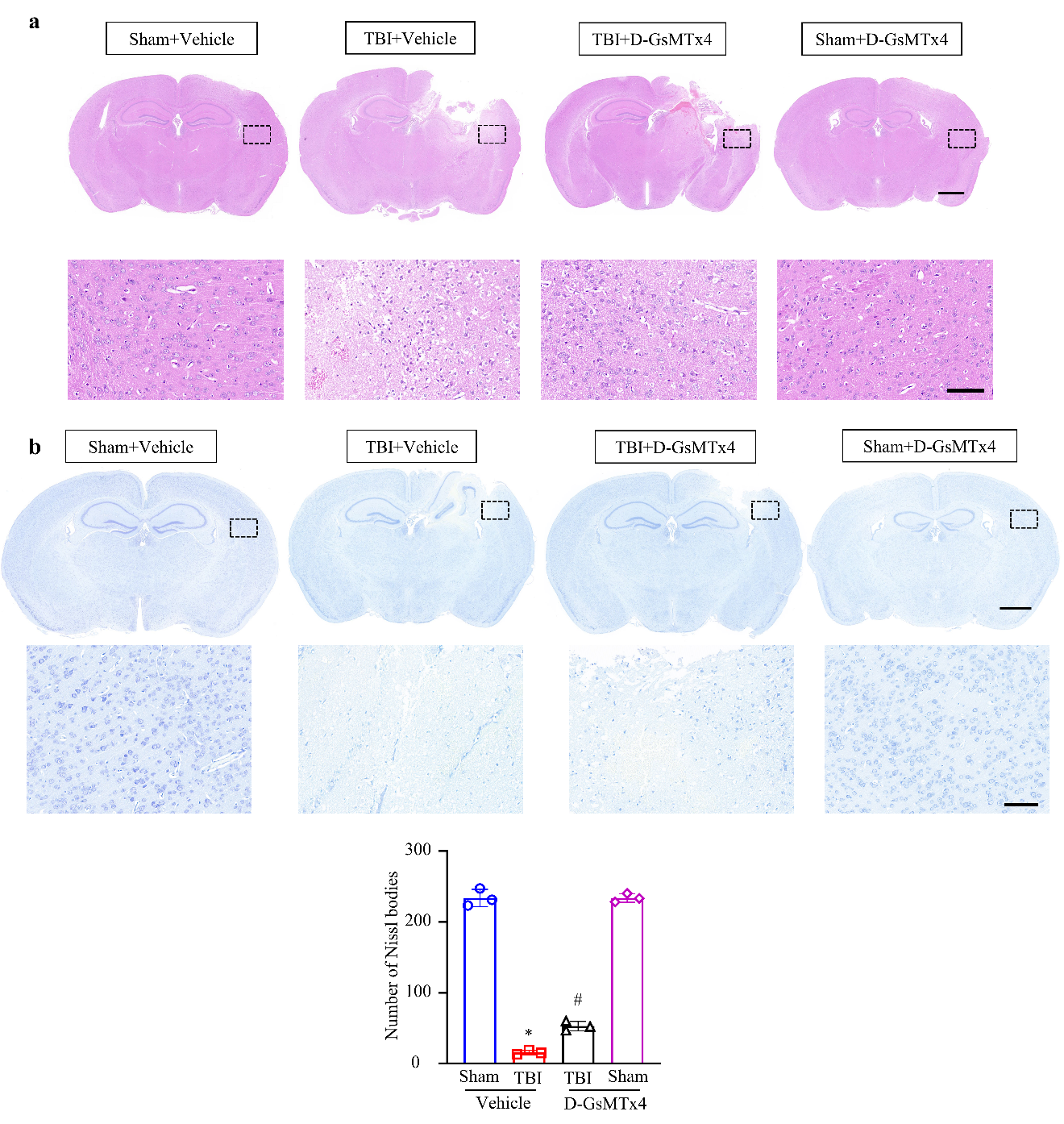


**Supplementary Fig. 3** Effect of administration of D-GsMTx4 or vehicle on brain tissue on day 3. (a) Representative HE-stained brain sections from the different treatment groups. Top: images of entire brain sections. Scale bar: 1000 µm. Bottom: magnification of the photographs in the top panel. Scale bar: 100 µm. (b) Representative Nissl-stained brain sections from the different treatment groups. Top: images of entire brain sections. Scale bar: 1000 µm. Bottom: magnification of the photographs in the top panel. Scale bar: 100 µm. n=3. One-way ANOVA followed by Bonferroni’s post hoc test. *P < 0.05 versus the Sham + NC group. #P < 0.05 versus the TBI + NC group.
